# Combined Effects of Aldehyde Dehydrogenase Variants and Maternal Mitochondrial Genes on Alcohol Consumption

**Published:** 2006

**Authors:** Yedy Israel, María E. Quintanilla, Amalia Sapag, Lutske Tampier

**Affiliations:** Yedy Israel, Ph.D., is a professor in the Department of Pharmacological and Toxicological Chemistry and the Department of Molecular and Clinical Pharmacology at the University of Chile, Santiago, Chile, and an adjunct professor in the Department of Pathology, Anatomy and Cell Biology at Thomas Jefferson University, Philadelphia, Pennsylvania. María E. Quintanilla, M.Sc., and Lutske Tampier, Pharm.D., are associ ate professors in the Department of Molecular and Clinical Pharmacology at the University of Chile, Santiago, Chile. Amalia Sapag, Ph.D., is an assistant professor in the Department of Pharmacological and Toxicological Chemistry at the University of Chile, Santiago, Chile

**Keywords:** Alcohol and other drug (AOD) consumption, ethanol metabolism, liver, mitochondria, genetic theory of AOD use (AODU), Wistar rats, UChA rats, UChB rats, aldehyde dehydrogenase (ALDH), ALDH2, acetaldehyde, nicotinamide adenine dinucleotide (NAD), protective factors, chemical aversion

## Abstract

Two lines of rats bred to differ in their voluntary alcohol consumption—the alcohol-abstaining UChA rats and the alcohol-drinking UChB rats—differ in how effectively toxic acetaldehyde is removed during alcohol metabolism. UChB animals carry efficient variants of the aldehyde dehydrogenase 2 (ALDH2) genes and have active mitochondria, resulting in fast removal of acetaldehyde. UChA animals, in contrast, carry less efficient ALDH2 variants and less active mitochondria, which result in transient elevations of acetaldehyde levels after alcohol ingestion. Cross-breeding studies have demonstrated that the presence of active mitochondria inherited from UChB females can fully abolish the reduction of alcohol consumption associated with the presence of less efficient ALDH2 variants—a phenomenon known as epistasis. These and other findings suggest that mitochondrial activity during alcohol metabolism should be considered a new modulator of alcohol consumption not only in rats but also in other species, including humans.

Through selective breeding approaches, researchers have generated several pairs of mouse or rat strains that are derived from the same ancestor populations but differ substantially in their voluntary alcohol consumption. For example, investigators at the University of Chile generated the alcohol “abstainer” (UChA) and the alcohol “bibulous” (UChB) rat lines, both of which were derived from Wistar rats (Mardones and Segovia-Riquelme 1983). When given a choice between tap water and water containing 10 percent alcohol, rats of the UChB line increase their alcohol consumption, whereas UChA rats do not, indicating that alcohol is reinforcing for UChB rats. However, the alcohol intake of individual UChB animals varies greatly, whereas no such variation has been observed among UChA rats. Since their development, rats of these two lines have been bred continuously by selectively mating animals that either consume less than 1 gram of alcohol per kilogram of body weight per day (g/kg/day) for the UChA line or more than 4 to 5 g/kg/day for the UChB line.

Studies conducted in recent years have identified, at least partly, the genetic and biochemical differences between UChA and UChB rats that contribute to the different alcohol consumption levels of the two lines (for a full review, see Quintanilla et al. 2006). These investigations point to central roles for the enzyme aldehyde dehydrogenase (ALDH) and for certain molecules found in the animals’ mitochondria, all of which are involved in alcohol metabolism.

## Differences in the Genes Encoding ALDH

As described in other articles in this journal issue, during alcohol metabolism in the liver, the alcohol (chemically known as ethanol) is first converted into acetaldehyde, a noxious compound. In a second step that takes place in the liver cells’ mitochondria, acetaldehyde is converted into the nontoxic acetate. This reaction is mediated by ALDH and involves the transfer of hydrogen atoms from the acetaldehyde to a compound known as nicotinamide adenine dinucleotide (NAD^+^), resulting in the formation of reduced NAD^+^ (NADH^+^). During this reaction, ALDH interacts with both acetaldehyde and NAD^+^.

In both humans and laboratory animals, researchers have identified several types of ALDH (e.g., ALDH1, ALDH2, etc.). Moreover, Sapag and colleagues (2003) have demonstrated that several variants (i.e., alleles) of the gene encoding ALDH2 exist in rats and that the resulting ALDH2 molecules differ in how easily they can interact with NAD^+^ (i.e., in their affinity for NAD^+^) and in the maximal speed (V_max_) with which acetaldehyde is converted to acetate. In general, higher affinity for NAD^+^ and higher V_max_ result in a faster removal of acetaldehyde from the body.

Specifically, Sapag and colleagues (2003) identified the following alleles:

- The *Aldh2*^1^ allele, which encodes a highly efficient ALDH2 molecule with high affinity for NAD^+^ and high V_max_
- The *Aldh2*^2^ allele, which encodes a relatively inefficient ALDH2 molecule with low affinity for NAD^+^ and low V_max_
- The *Aldh2*^3^ allele, which encodes an intermediately efficient ALDH2 molecule with high affinity for NAD^+^ and low V_max_

**Figure 1:**
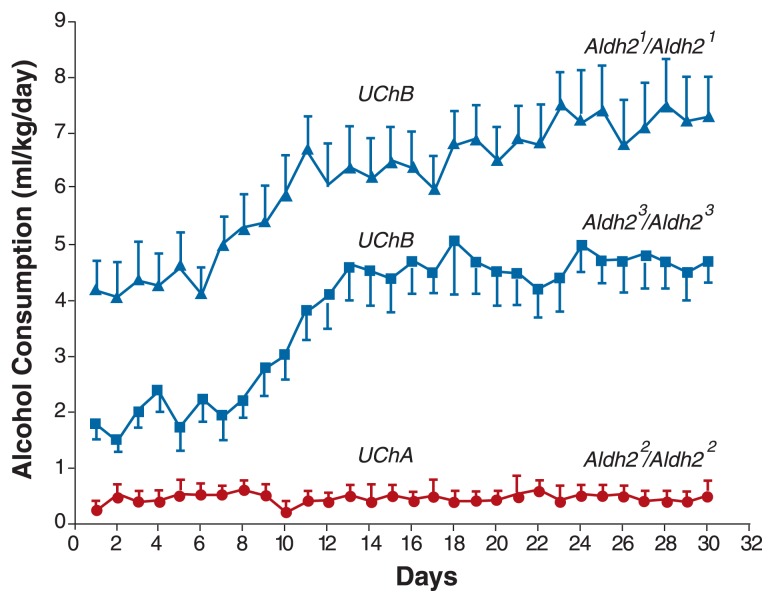
Voluntary ethanol consumption according to *Aldh2* alleles in UChB and UChA Wistar-derived rats. Alcohol-naïve rats of the UChA and UChB lines were allowed free access to 10 percent alcohol and tap water from two separate bottles for 30 days. The animals’ status with respect to *Aldh2* alleles (i.e., the *Aldh2* genotype) was determined before they received access to alcohol. The animals’ alcohol consumption was calculated daily and averaged. UChB animals drank significantly more alcohol than UChA animals. Moreover, UChB animals with an *Aldh2*^1^*/Aldh2*^1^ genotype consumed more alcohol than UChB animals with an *Aldh2*^3^*/Aldh2*^3^ genotype. SOURCE: Quintanilla et al. 2006.

The investigators found that animals of the alcohol-abstaining UChA line carry almost exclusively the inefficient *Aldh2*^2^ allele, whereas animals of the alcohol-drinking UChB line carry the more efficient *Aldh2*^1^ and *Aldh2*^3^ alleles. Furthermore, UChB animals carrying the highly efficient *Aldh2*^1^ allele differed in alcohol consumption from UChB animals carrying the less efficient *Aldh2*^3^ allele (see Figure 1). The kinetic differences between the enzymes encoded by *Aldh2*^1^ and *Aldh2*^3^ explained one-third of the differences in alcohol intake among UChB rats.

## Role of Mitochondrial Activity

Additional studies identified differences between UChA and UChB rats in the animals’ mitochondria. For acetaldehyde to be effectively turned into acetate, the NADH^+^ that also is generated during the reaction must be converted back into NAD^+^, a process known as oxidation. Quintanilla and colleagues (2005*a*) demonstrated that the mitochondria of UChA rats are less active in oxidizing NADH than the mitochondria of UChB rats.

The researchers then examined the interactions between efficient and inefficient *Aldh2* alleles on the one hand and active and less active mitochondria on the other hand by selectively mating UChA and UChB animals and their offspring. For these experiments it is important to know that the genes encoding the proteins that determine mitochondrial activity are inherited only from the mother. Thus, if a female with active mitochondria is mated with a male with inactive mitochondria, all the offspring will have active mitochondria. Conversely, if a female with inactive mitochondria is mated with a male with active mitochondria, all of the offspring will have inactive mitochondria.

**Figure 2:**
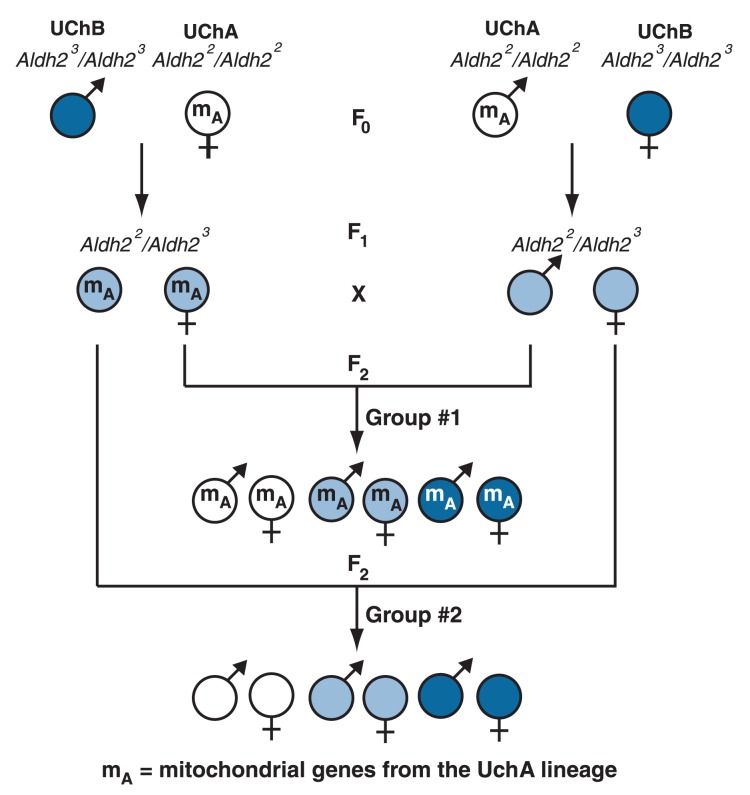
Breeding scheme used to determine the role of *Aldh2* alleles and mitochondrial activity on alcohol consumption. In the first stage, UChB males (*Aldh2* ^3^*/Aldh2* ^3^ genotype [blue circles]) were mated with UChA females *(Aldh2* ^2^*/Aldh2* ^2^ genotype [white circles]) and vice versa. The resulting F_1_ animals all had the *Aldh2* ^3^*/Aldh2* ^3^ genotype (light blue circles), but half of them carried the less active UChA mitochondria (denoted as m_A_), and half carried the active UChB mitochondria. F_1_ animals then were mated with each other, such that an animal with UChA mitochondria was mated with an animal with UChB mitochondria. The resulting F_2_ animals again fell into two groups—those with UChA mitochondria and those with UChB mitochondria. In each group, there were males and females with any of the three *Aldh2* genotypes.

For their experiments, Quintanilla and colleagues (2005a) mated original (or F_0_) UChA rats carrying two inefficient *Aldh2*^2^ alleles (*Aldh2*^2^/*Aldh2*^2^) and F_0_ UChB rats carrying two efficient *Aldh2*^3^ alleles (*Aldh2*^3^/*Aldh2*^3^). The resulting offspring (known as F_1_ animals) were then mated amongst each other to generate F_2_ animals, in which all genes had been “shuffled.” Furthermore, the researchers used a breeding design that allowed them to trace the origin of the maternal mitochondrial genes (UChA or UChB) (see Figure 2). As a result of this strategy, the F_2_ animals had the following characteristics:

- They carried one of three possible combinations of *Aldh2* genes— namely *Aldh2*^2^*/Aldh2*^2^, *Aldh2*^2^*/Aldh2*^3^, or *Aldh2*^3^*/Aldh2*^3^
- They carried either “abstainer” mitochondrial genes (if the female in the F_0_ generation was UChA) or “drinker” mitochondrial genes (if the female in the F_0_ generation was UChB).

The study found that the combination of a less efficient ALDH2 (i.e., the presence of two *Aldh2*^2^ alleles) and less active mitochondria (i.e., the presence of UChA-derived mitochondria) potentiated each other and accounted for 60 to 70 percent of the lower voluntary alcohol consumption of UChA versus UChB rats. Furthermore, the alcohol consumption of *Aldh2*^2^*/Aldh2*^2^ animals was low only when they carried the less active UChA mitochondrial genes but not when they carried the more active UChB mitochondrial genes. This observation indicates that the presence of some genes (i.e., the genes responsible for the more active mitochondria) abolishes the effects of other genes (i.e., the *Aldh2*^2^ genes)—a phenomenon known as epistasis. In fact, this is the first case of epistasis described in the alcohol consumption/alcohol dependence literature. This interaction is gender related because the mitochondrial genes are inherited only from the mother.

The reverse was true for animals carrying the efficient *Aldh2*^3^*/Aldh2*^3^ genes: these animals consumed large amounts of alcohol regardless of the activity of the mitochondria. Thus, it appears that in these animals the presence of two *Aldh2*^3^*/Aldh2*^3^ alleles abolished the effect of genes responsible for a lower (UChA) mitochondrial activity (Quintanilla et al. 2005*a*).

### Genes Determining Mitochondrial Activity

Additional studies have demonstrated that the reduced activity of mitochondria of UChA animals results from a defect in a mitochondrial protein complex called Complex I (NADH-ubiquinone oxidoreductase). The function of this protein complex is to oxidize NADH^+^, thereby regenerating NAD^+^. Complex I consists of numerous protein components (i.e., subunits), including seven proteins encoded by (maternal) mitochondrial genes. Sapag and colleagues (2006) demonstrated that one of the Complex I subunits, the maternally inherited protein ND2, differs in five protein building blocks (i.e., amino acids) between UChA and UChB rats. The *Nd2* allele found in UChA rats differs from all other *Nd2* alleles reported in the literature; moreover, this specific allele was found in every UChA rat studied.

## Impact of ALDH Status and Mitochondrial Activity on Acetaldehyde Levels

It is well established that accumulation of high acetaldehyde levels in the blood has an aversive effect—that is, it discourages an individual from drinking more alcohol. Therefore, Quintanilla and colleagues (2005*b*) examined whether blood acetaldehyde levels differed between UChA and UChB rats. These studies found that the normally nondrinking UChA animals exhibit a blood “acetaldehyde burst” within 5 to 15 minutes of alcohol administration, with acetaldehyde levels five times higher than those found in UChB animals. At later times, however, when alcohol levels in the body are still high, the acetaldehyde levels measured in UChA and UChB rats were comparable.

Based on information on the factors that determine the rate with which alcohol metabolism proceeds, it has been postulated that acetaldehyde generation is highest shortly after alcohol metabolism has been initiated, when NADH^+^ levels in the cell are low, and decreases at later times, when NADH^+^ levels have been elevated by alcohol metabolism itself. The high acetaldehyde levels initially generated could be metabolized more efficiently in UChB animals with their highly efficient ALDH2 enzyme and the fully functional mitochondrial Complex I system than in UChA animals with their ineffective ALDH2 enzyme and dysfunctional Complex I system. As a result, a transient acetaldehyde burst would occur only in UChA animals.

Researchers also have tried to reduce the alcohol consumption of UChB rats by reducing the animals’ ALDH2 activity. To this end, the investigators injected the animals with a harmless virus (i.e., an adenovirus) carrying an antisense gene—a molecule that can bind to the animals’ *Aldh2*^1^ gene product (mRNA), thereby preventing the information encoded by the *Aldh2* gene from being converted into a protein. The researchers found that following a single intravenous injection of this virus, the animals’ alcohol consumption was reduced by 50 percent for 1 month (Ocaranza et al. 2006; also see Karahanian et al. 2005).

Inhibition of ALDH2 activity also occurs during treatment with the medication disulfiram, which is prescribed to about 10 percent of alcohol-dependent patients in the United States (Mark et al. 2003). Disulfiram inhibits all types of ALDH in all organs that have the enzyme and can eliminate any toxic acetaldehyde that reaches them via the blood (Oyama et al. 2005; Morita et al. 2005). In contrast, the adenovirus used in the experiments described above delivers its genes mostly to the liver (but not the brain), reducing ALDH2 activity primarily in this organ. Localized reduction, mainly of liver ALDH2, allows other tissues to retain their ability to detoxify any potentially harmful acetaldehyde reaching them. Such an approach, which mimics the “acetaldehyde burst” phenomenon by partially inhibiting hepatic ALDH2 and results in elevation of acetaldehyde levels only for the short time needed to induce aversion to alcohol, might constitute an improved alcoholism therapy compared with disulfiram.

## Summary

The studies discussed here indicate that mutations which render ALDH2 less efficient, combined with less active mitochondria, result in a blood acetaldehyde burst and are associated with an aversion to alcohol. The studies suggest that the ability of mitochondria to oxidize NADH^+^ should be considered a new modulator of alcohol consumption not only in rodents but also in other species, including humans. Moreover, the results indicate that future research on treatment approaches based on aversion to alcohol might benefit from exploring whether a blood acetaldehyde burst also occurs in humans shortly after alcohol ingestion.
